# Advanced Child Tax Credit Monthly Payments and Substance Use Among US Parents

**DOI:** 10.1001/jamahealthforum.2024.4699

**Published:** 2025-01-03

**Authors:** J. Travis Donahoe, Brittany L. Brown-Podgorski, Sabin Gaire, Elizabeth E. Krans, Marian Jarlenski

**Affiliations:** 1 Department of Health Policy and Management, University of Pittsburgh School of Public Health, Pittsburgh, Pennsylvania; 2 Department of Obstetrics, Gynecology, and Reproductive Services, University of Pittsburgh School of Medicine, Magee-Women’s Research Institute, Pittsburgh, Pennsylvania

## Abstract

**Importance:**

2021 Advance child tax credit (ACTC) monthly payments were associated with reduced US child poverty rates; however, policymakers have expressed concerns that permanent adoption would increase parental substance use.

**Objective:**

To assess whether 2021 ACTC monthly payments were temporally associated with changes in substance use among parents compared with adults without children.

**Design, Setting, and Participants:**

The primary sample included adults aged 18 to 64 years who responded to the National Survey on Drug Use and Health in 2021. Difference-in-differences models were used to test whether substance use changed for parents compared with adults without children after ACTC monthly payments went into effect. Additional National Survey on Drug Use and Health data from 2018 to 2020 were used to assess pretrends in substance use for parents and adults without children. Analyses were survey weighted and conducted from September 2023 to November 2024. The treatment group was defined as adults with dependent children in the home who would have been eligible for the tax credit from July to December 2021. Adults without children, who would not have been eligible for the tax credit, comprised the comparison group.

**Main Outcomes and Measures:**

Outcomes included binary measures of any self-reported use of tobacco, alcohol, cannabis, and illicit substances (eg, cocaine, opioids, or other stimulants or sedatives) during the previous 30 days; counts of the number of days of use of tobacco, alcohol, cannabis, and illicit substances during the previous 30 days among people who used these substances; and counts of the number of cigarettes and alcoholic beverages consumed during the previous 30 days among people who used these substances.

**Results:**

Of 41 853 adults, 17 308 were parents and 24 545 were adults without children. ACTC monthly payment implementation was associated with a −4.3–percentage point (95% CI, −6.6 to −2.0) decline in the probability of using tobacco during the previous 30 days for parents compared with adults without children. Among parents who smoked, payments were associated with a −46.8–percentage point (95% CI, −93.1 to −0.5) decline in the number of cigarettes smoked during the previous 30 days. Estimated changes in the probability, frequency, and quantity of other substance use (alcohol, cannabis, and illicit substances) for parents were null and not significant.

**Conclusions:**

The study results suggest that 2021 ACTC monthly payments were not associated with increased parental substance use.

## Key Points

**Question:**

Were 2021 advance child tax credit monthly payments associated with changes in substance use among parents?

**Finding:**

In this difference-in-differences study of 41 853 adults, 2021 advance child tax credit monthly payments were temporally associated with reduced tobacco use among parents compared with adults without children (who were not eligible). There were no significant associations between advance child tax credit payments and changes in the probability or frequency of alcohol, cannabis, or illicit substance use among parents.

**Meaning:**

The study results do not support concerns that providing increased financial support to parents via the advance child tax credit would be associated with increased substance use.

## Teaser

This difference-in-differences analysis examines whether 2021 advance child tax credit monthly payments were associated with changes in substance use among parents compared with adults without children.

## Introduction

The advance child tax credit (ACTC) passed by the US Congress in 2021 expanded the scope of the child tax credit from $2000 to $3000 for children older than 6 years and $2000 to $3600 for children younger than 6 years.^1^ In addition, the law raised the age limit for children who were eligible for the tax credit from 16 to 17 years, made the tax credit fully refundable (so it was available to families who did not file or owe taxes), and mandated that half of the credit be dispersed monthly via direct deposit or check. Eligibility was verified from existing governmental records, resulting in payments being sent to nearly all eligible families. This created a policy design similar to unconditional cash transfer programs.^2^

This expansion was unprecedented in scope for US family assistance programs. Data from the US Census Bureau showed that the ACTC was associated with a reduction in childhood poverty rates to their lowest point on record.^3^ However, when the monthly payments expired in 2022, Congress did not extend the policy, and childhood poverty rebounded.^4^

A long-standing concern of critics of unconditional cash transfer programs like the ACTC is that they may be associated with increased substance use. In 2021, news outlets reported this concern from policymakers who voted against extending the ACTC in 2022.^5^ More broadly, concerns about cash transfer programs enabling drug use have been pervasive enough that several states require mandatory urine drug screenings to participate in cash assistance programs.^6^ Historically, such concerns have also been racialized and have had negative implications for social safety net expansions.^7,8^

The potential ways that monthly cash transfers might be associated with substance use are unclear. Aggregate demand for normal consumer goods increases with higher incomes; however, whether a good is normal and the magnitude of the association for any specific good is unclear and depends on how peoples' preferences change with income.^9^ Further, many people use substances (particularly tobacco) as a response to stress,^10^ making it possible that increased financial supports could decrease use through alleviating financial stressors. Lastly, it is also possible for there not to be any association between cash transfers and substance use. The inability to control one’s level of substance use is a defining feature of substance use disorders^11^; thus, for people who physiologically depend on substances, one might expect similar levels of use regardless of income.

The research on cash transfers and substance use is mixed. Several studies have found small increases in adverse substance use outcomes (eg, overdose hospitalizations) that were associated with the timing of public assistance income receipt.^12,13,14^ However, it is unknown whether these findings are due to increased substance use or intertemporal substitution.^15^ Further, in contrast to these studies, a recent randomized clinical trial of a $313 monthly cash benefit to mothers found that these transfers had no effect on self-reported substance use or alcohol and tobacco purchases.^16^ Another randomized study of cash benefits to individuals with low incomes in Chelsea, Massachusetts, found that the benefits reduced substance use–related emergency department visits.^17^

To our knowledge, few studies have used nationally representative samples to evaluate the association between unconditional cash transfer programs and substance use. To address this gap, we used national data on substance use to investigate the temporal association between ACTC monthly payments and parental substance use. To our knowledge, this is the first study of the association between implementation of a national unconditional cash transfer program and substance use in the US.

## Methods

### Data Source and Population

We used data from the National Survey of Drug Use and Health (NSDUH). NSDUH is a nationally representative survey on substance use among noninstitutionalized people 12 years and older in the US. Our primary analysis was limited to data from 2021 due to survey methods changes between 2020 and 2021 and a lack of quarterly data in 2020. This primary sample in 2021 consisted of 41 853 adults aged 18 to 64 years and was analyzed with survey weights so that they were representative of all US adults. A total of 17 308 respondents were parents (defined as reporting at least 1 dependent child younger than 19 years in the household) and 24 545 were not parents (who did not report dependent children). In additional analyses of pre-ACTC trends (described later), we incorporated data on all adults (N = 102 084) from 2018 to 2020. We followed the Strengthening the Reporting of Observational Studies in Epidemiology (STROBE) reporting guideline checklist for cross-sectional studies. No institutional board review or informed consent waiver was required for this analysis of publicly available and deidentified data.

### Measures and Outcomes

#### Person-Level Characteristics

In the NSDUH, age was categorized as 18 to 25, 26 to 49, and 50 to 64 years. Sex was categorized in the NSDUH as male and female. Self-reported race and ethnicity were categorized as Hispanic; non-Hispanic Alaskan, Hawaiian, Native American, or Pacific Islander; non-Hispanic Asian; non-Hispanic Black; non-Hispanic multiracial; and non-Hispanic White. Marital status was categorized as married, never married, and widowed, separated, or divorced, and education was dichotomized by 4-year college completion.

#### Child Tax Credit

The ACTC monthly payments were sent to nearly all eligible families from July to December 2021. Two-parent families with incomes up to $150 000 were eligible for tax credits of $3600 for each child younger than 6 years and $3000 for each child aged 6 to 17 years; at incomes between $150 000 and $400 000, the tax credits phased down to a minimum of $2000 per child and to $0 at incomes greater than $400 000.^18^ Because income eligibility thresholds for the child tax credit were high, we considered all parents treated by the ACTC monthly payments when they were distributed.

#### Substance Use

We examined 3 types of outcomes: (1) any substance use, (2) frequency of substance use among people who use substances, and (3) quantity of substance use among people who use substances. Any substance use was a binary measure defined as the self-reported use of alcohol, cannabis, tobacco, or illicit substances (cocaine, opioids, or other stimulants or sedatives) during the previous 30 days. Frequency of substance use was defined as the number of days of alcohol, cannabis, tobacco, or illicit substance use during the previous 30 days among people who reported using these substances. Quantity of substance use was defined as the number of days using tobacco (alcohol) multiplied by the number of cigarettes smoked (alcoholic beverages consumed) on days respondents used tobacco (alcohol). The quantity variables were also restricted to respondents who reported use. The NSDUH variable that quantified the number of cigarettes smoked used a range (eg, 2 to 5 cigarettes). We converted these to the midpoint of each range (eg, 3.5 in the case of 2 to 5 cigarettes) for regression analyses. The number of cigarettes was also top coded at 35.

Missing data (ranging from 0.02%-1.% across study outcomes) were statistically imputed, as described in NSDUH documentation.^19^ To generate prevalence estimates that accounted for differential nonresponse, we included individuals with statistically imputed outcomes. Results were not sensitive to this analytical choice.

### Statistical Analysis

The primary analysis used difference-in-differences (DiD) models to estimate the association of ACTC monthly payments with parental substance use. Linear regression models were fit for each outcome and included a binary variable for parental status, a binary variable for when ACTC monthly payments were in effect (July-December 2021), and person-level covariates. An interaction term for the binary parent and ACTC variables was used to capture the association of parental substance use with ACTC monthly payments.

#### Examining Pretrends

Our analysis relied on the assumption that trends in outcomes for parents and adults without children would have been parallel if not for the ACTC monthly payments. We assessed the plausibility of this assumption by examining trends for parents vs adults without children before the ACTC was implemented and testing for pretrends using event study regressions that interacted parental status with each period compared with when the ACTC monthly payments were implemented. This was complicated by survey methods changes to NSDUH in 2021 and a lack of quarterly data in 2020. Thus, we interpreted these models that spanned the period from 2020 to 2021 cautiously and used data from 2021 only in our primary analysis.

#### Robustness Analyses

Across all households, ACTC monthly payments may have been small compared with income, leading to small associations that are difficult to detect as an average population association. To assess robustness to larger income changes, we repeated our analyses, limiting the sample to adults with incomes less than 200% of the federal poverty level (FPL), for whom ACTC payments represented a sizable portion of monthly income. Additionally, we estimated continuous DiD models that used the number of dependent children in a household to define treatment status, as monthly payments were allocated on a per-child basis.

To identify adults with incomes less than 200% of the FPL, we used NSDUH measures of household income and the number of children in each household. Poverty levels were obtained for 2021 from the US Department of Health and Human Services poverty guidelines. Income is reported in brackets in NSDUH. To calculate income as a percentage of FPL, we used the midpoint income for each income bracket and the top coded value of $75 000 for all households that earned more than that income. The number of children in each household was also top coded by NSDUH at 3.

Estimating equations are provided in the eAppendix in Supplement 1. All results used survey weights and accounted for clustered sampling. Analyses were implemented from September 2023 to November 2024 in R, version 4.3.0 (R Foundation), and Stata, version 17 (StataCorp). Statistical significance was assessed at the 5% level.

## Results

### Descriptive Statistics

The study population consisted of 41 853 adults (17 308 parents; 24 545 adults without children). Person-level characteristics and study outcomes, as stratified by parental status and timing in comparison with ACTC monthly payments, are reported in Table 1. Compared with adults without children, parents were older, more likely to be married, and female. Parents were also more likely to be Hispanic and less likely to be non-Hispanic White. These differences were similar in the pre-ACTC and post-ACTC monthly payment periods. During the pre-ACTC period, parents reported being less likely to use tobacco, alcohol, cannabis, or illicit substances. Among those who used substances, the frequency and quantity of use were similar for parents and adults without children.

**Table 1.** Descriptive Characteristics of Parents and People Without Children Before vs After Advanced Child Tax Credit (ACTC) Monthly Payments^a^

Characteristic	% (SE)			
	Pre-ACTC monthly payments (January-June 2021)		Post-ACTC monthly payments (July-December 2021)	
	Parents (n = 8460)	People without children (n = 12 133)	Parents (n = 8848)	People without children (n = 12 412)
Covariates				
Age, y				
18-25	14.4 (0.7)	18.6 (0.6)	14.6 (0.6)	18.3 (0.6)
26-49	69.6 (1.2)	39.7 (0.9)	69.7 (1.1)	38.8 (0.9)
50-64	16.0 (1.1)	41.7 (1.1)	15.6 (1.2)	43.0 (1.1)
Marital status				
Married	60.1 (1.2)	37.8 (1.0)	57.5 (0.9)	36.1 (1.0)
Never married	26.9 (1.0)	43.9 (0.9)	29.5 (0.8)	45.3 (1.1)
Widowed, separated, or divorced	12.9 (0.8)	18.3 (0.8)	13.0 (0.6)	18.6 (0.7)
Education < college degree	68.4 (1.2)	66.5 (1.0)	70.6 (1.2)	69.0 (1.0)
Sex				
Female	55.4 (0.9)	48.1 (1.0)	56.1 (1.0)	45.6 (0.8)
Male	44.6 (0.9)	51.9 (1.0)	43.9 (0.9)	54.4 (0.8)
Race and ethnicity				
Hispanic	24.9 (1.6)	15.3 (0.9)	23.3 (1.2)	16.3 (0.9)
Non-Hispanic Asian	5.9 (0.4)	6.1 (0.5)	7.1 (0.7)	5.8 (0.4)
Non-Hispanic Black	12.9 (0.8)	13.4 (0.8)	13.1 (0.8)	12.4 (0.6)
Non-Hispanic multiracial	1.7 (0.2)	2.1 (0.2)	2.0 (0.3)	2.2 (0.2)
Non-Hispanic Alaskan, Hawaiian, Native American, or Pacific Islander	1.2 (0.3)	0.7 (0.1)	1.1 (0.2)	1.4 (0.2)
Non-Hispanic White	53.3 (1.3)	62.4 (1.0)	53.4 (1.7)	61.9 (1.1)
Outcomes during the previous 30 d				
Probability of any use				
Any cigarette use	17.6 (0.7)	18.9 (0.7)	16.0 (0.7)	21.4 (0.8)
Any alcohol use	52.2 (1.2)	56.2 (1.0)	51.6 (1.2)	54.9 (0.9)
Any cannabis use	12.3 (0.6)	17.3 (0.9)	13.9 (0.6)	19.1 (1.0)
Any illicit substance use	1.7 (0.2)	3.8 (0.3)	2.3 (0.3)	3.7 (0.3)
No. of days of use, mean (SE)				
Cigarettes	22.4 (0.6)	22.5 (0.4)	22.3 (0.5)	23.0 (0.4)
Alcohol	7.8 (0.2)	8.6 (0.2)	7.4 (0.2)	8.8 (0.2)
Cannabis	17.9 (0.5)	16.3 (0.5)	17.2 (0.5)	16.1 (0.4)
Illicit substances	10.6 (1.3)	9.8 (0.9)	8.3 (1.1)	11.7 (1.2)
Quantity of use, mean (SE)				
No. of cigarettes	295.8 (16.1)	287.1 (12.2)	267.0 (13.4)	313.1 (12.6)
No. of alcoholic beverages	24.9 (1.8)	28.1 (1.3)	22.1 (1.2)	31.8 (3.1)

^a^ Data are from the National Survey on Drug Use and Health from all adults aged 18 to 64 years. Results used survey weights to represent the target population of all US parents and people without children aged 18 to 64 years.

### Unadjusted Trends

Unadjusted trends in the probability of substance use and frequency of substance use during the previous 30 days are presented for parents and adults without children from 2018 to 2021 in the Figure. Adults without children reported higher levels of substance use for each type of substance than parents; however, trends for parents compared with adults without children were visually similar during the pre-ACTC period. For the frequency of use variables, the trends and levels of substance use for parents and adults without children were similar during the pre-ACTC period. eFigure 1 in Supplement 1 shows that trends in the quantity of substance use for tobacco and alcohol were also parallel for adults without children and parents before ACTC monthly payments. We formally tested for pretrends, as described later in the article. For tobacco, the probability (Figure) and quantity of use (eFigure 1 in Supplement 1) declined for parents (compared with adults without children) when ACTC monthly payments were in effect. For other outcomes, trends remained parallel for parents and adults without children during ACTC monthly payments.

**Figure.**
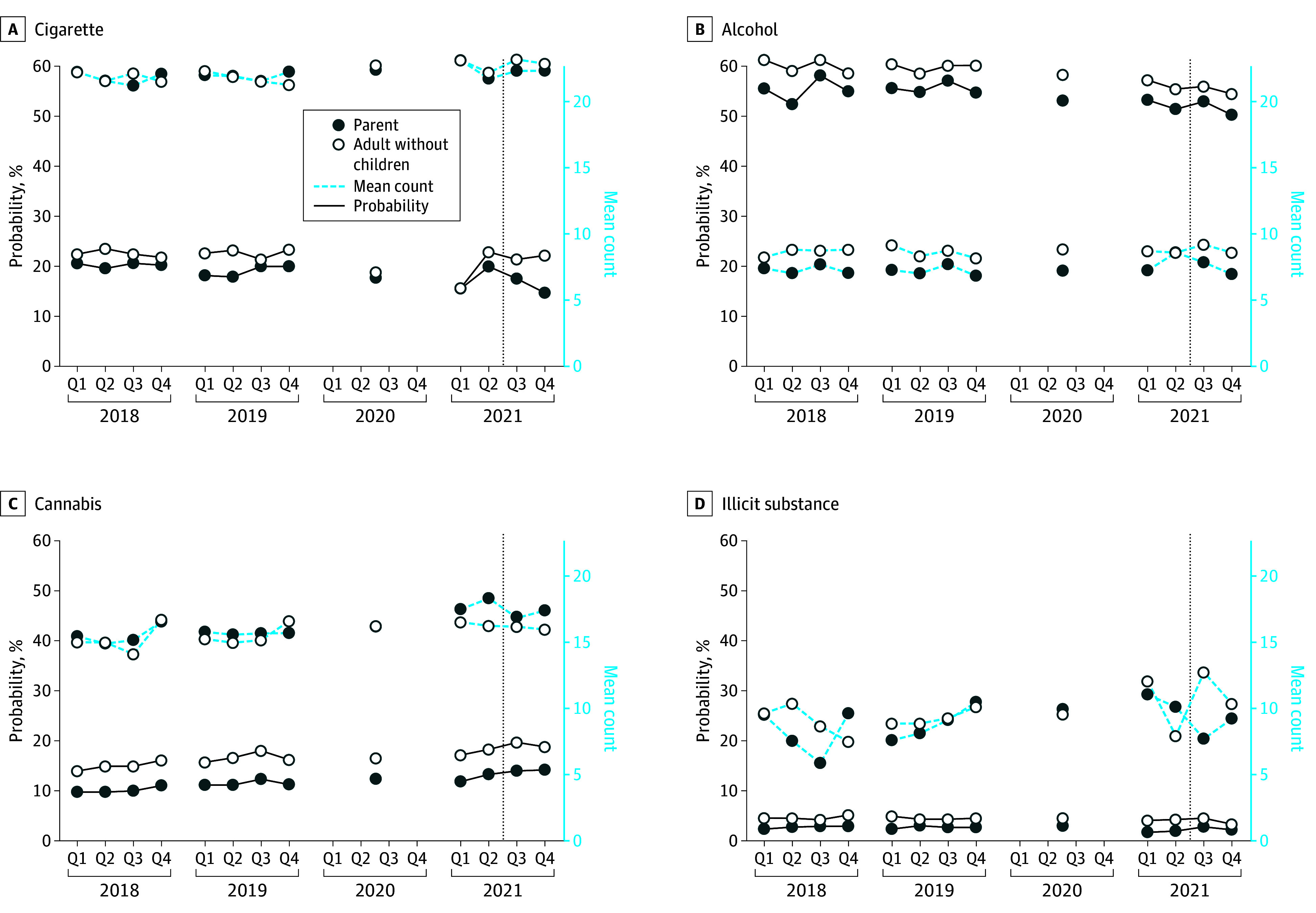
Trends in Study Outcomes for Parents vs People Without Children From 2018 to 2021 Data are from the National Survey on Drug Use and Health among all adults aged 18 to 64 years from 2018 to 2021. The vertical line indicates when advanced child tax credit monthly payments were sent out from July to December 2021. Quarterly data were not available for 2020. Q indicates quarter.

### Association With the Probability, Frequency, and Quantity of Substance Use

Estimates of the association between ACTC monthly payments and substance use are reported in Table 2. We observed a −4.3–percentage point (pp; 95% CI,−6.6 to −2.0) reduction in the probability of any tobacco use during the previous 30 days among parents (compared with the change for adults without children) when ACTC monthly payments were in effect. For alcohol, cannabis, and illicit substances, the association between ACTC monthly payments and substance use was null. Confidence intervals ruled out positive associations between ACTC monthly payments and the probability of use of these substances of more than 5.3, 2.9, and 1.9 pp with 95% confidence.

**Table 2.** Difference-in-Differences Estimate of the Association Between Advanced Child Tax Credit Monthly Payments and Parental Substance Use^a^

Outcome variable during previous 30 d	No. of individuals	Association with ATC monthly payments, pp (95% CI)
Probability of any use		
Tobacco	41 853	−4.3 (−6.6 to −2.0)
Alcohol	41 853	1.0 (−3.3 to 5.3)
Cannabis	41 853	−0.2 (−3.3 to 2.9)
Illicit substances	41 853	0.8 (−0.3 to 1.9)
No. of days of use		
Tobacco	6813	−0.5 (−2.4 to 1.5)
Alcohol	23 319	−0.7 (−1.6 to 0.1)
Cannabis	7579	−0.6 (−2.5 to 1.5)
Illicit substances	1380	−4.8 (−9.9 to 0.3)
Quantity of use		
No. of cigarettes	6813	−46.8 (−93.1 to −0.5)
No. of alcoholic drinks	23 319	−6.4 (−15.0 to 2.2)

Abbreviations: ATC, advance tax credit; pp, percentage point.

^a^ Data are from the National Survey on Drug Use and Health, among all adults aged 18 to 64 years (N = 41 853). Results are from difference-in-differences linear regression models. All results use survey weights and report confidence intervals that were adjusted for clustered sampling.

Among people who used substances, the association between ACTC monthly payments and the number of days of use during the previous 30 days was null for tobacco, alcohol, cannabis, and illicit substances. Furthermore, the 95% CIs ruled out even small positive associations between ACTC monthly payments and the number of days of substance use with 95% confidence.

In terms of the quantity of substance use, ACTC monthly payments were associated with parents smoking −46.8 (95% CI, −93.1 to −0.5) fewer cigarettes during the previous 30 days. eFigure 2 in Supplement 1 shows that this is due to parents being less likely to smoke very high quantities of cigarettes daily when ACTC monthly payments were in effect. The association with the number of alcoholic beverages consumed during the previous 30 days was also negative but not statistically significant. Confidence intervals ruled out positive associations with alcoholic beverage use of greater than 2.2 drinks during the previous 30 days with 95% confidence.

### Pretrends Tests From Event Study Models

Event study plots for each outcome are presented in the eFigures 3 to 5 in Supplement 1. Each panel presents the *P* value from a hypothesis test that the interaction between parental status and each quarter before ACTC monthly payments were in effect are jointly equal to 0. We generally observed parallel pretrends and did not reject parallel pretrends for any outcome at a statistical significance level of 5%. Consistent with our primary DiD results, we also observed declines in the probability of tobacco use (eFigure 3 in Supplement 1) and the quantity of cigarettes smoked during the previous 30 days (eFigure 5 in Supplement 1) for parents (compared with adults with children) after ACTC monthly payments went into effect.

### Robustness to Larger Relative and Absolute Income Changes

Results for the sample whose incomes were less than 200% of FPL are reported in Table 3. Compared with adults without children, the probability of any tobacco use declined by −6.1 pp (95% CI, −10.8 to −1.4) for parents with incomes less than 200% of the FPL when ACTC monthly payments were in effect. There was also a large (albeit statistically imprecise due to the smaller sample size) negative association between payments and the quantity of cigarettes smoked. Associations between ACTC payments and other outcomes were null. Results that allowed for a linear association between the number of children and ACTC monthly payments are also presented in Table 3. We again observed a negative association between exposure to ACTC monthly payments and the probability of previous 30-day tobacco use. We also observed a negative association between payments and frequency of alcohol use. The estimates implied that each additional child was associated with −1.5 pp (95% CI, −2.8 to −0.2) lower probability of using tobacco and, among parents who consumed alcohol, −0.4 (95% CI, −0.9 to −0.0003) fewer days of alcohol use during the previous 30 days when ACTC payments were in effect. We did not observe significant and positive associations between payments and substance use for any outcome.

**Table 3.** Association of Parental Substance Use With Advanced Child Tax Credit (ATC) Monthly Payments Among Adults With Incomes Less Than 200% of the Federal Poverty Line (FPL) and Exposure as Defined by Number of Children^a^

Outcome variable during previous 30 d	Association with ATC monthly payments, pp (95% CI)			
	Parent status among adults with incomes at or less than 200% of FPL	No.	No. of children among all adults	No.
Probability of any use				
Tobacco	−6.1 (−10.8 to −1.4)	15 324	−1.5 (−2.8 to −0.2)	41 853
Alcohol	0.3 (−6.0 to 6.5)	15 324	0.4 (−1.5 to 2.4)	41 853
Cannabis	0.5 (−4.1 to 5.1)	15 324	0.3 (−1.2 to 1.8)	41 853
Illicit substances	−0.1 (−2.0 to 1.8)	15 324	0.4 (−0.01 to 1.0)	41 853
No. of days of use				
Tobacco	−0.1 (−2.2 to 2.1)	3705	−0.04 (−1.0 to 0.9)	6813
Alcohol	−0.2 (−1.6 to 1.2)	6980	−0.4 (−0.9 to −0.0003)	23 319
Cannabis	1.1 (−2.1 to 4.3)	3364	−0.3 (−1.2 to 0.7)	7579
Illicit substances	−1.4 (−9.5 to 6.6)	678	−2.1 (−4.6 to 0.5)	1380
Quantity of use				
No. of cigarettes	−47.5 (−119.3 to 24.3)	3705	−15.5 (−36.0 to 5.0)	6813
No. of alcoholic drinks	−2.6 (−18.2 to 12.9)	6980	−3.0 (−7.2 to 1.1)	23 319

Abbreviation: pp, percentage point.

^a^ Data are from the National Survey on Drug Use and Health among all adults aged 18 to 64 years with incomes less than 200% of the FPL in the first model and all adults in the second model. Results are from difference-in-differences linear regressions. All results use survey weights and report confidence intervals that were adjusted for clustered sampling.

## Discussion

In this DiD study, we examined the temporal association of 2021 ACTC monthly payments with several parental substance use outcomes. We found that the ACTC monthly payments were associated with reductions in the probability of parents using tobacco and the quantities of cigarettes that they smoked during the previous 30 days. The magnitude of these associations was large and equated to 24% and 16% reductions, respectively, compared with parents’ preperiod mean rates of tobacco use and cigarette smoking. For other substance use outcomes, we observed null associations between ACTC monthly payments and substance use that were relatively precise (ie, had confidence intervals that ruled out small increases).

There are several explanations for our findings that ACTC payments were not associated with increased substance use. First, multiple factors contribute to the prevalence of substance use in a population, such as socioenvironmental influences and individual-level psychosocial and genetic factors.^20^ As such, it is not likely that an increase in monthly income alone would be associated with more people deciding to use substances. Second, among parents already using substances and who may have a substance use disorder, changes in substance use behaviors are also associated with their level of physiological dependence and many individual and socioenvironmental factors unrelated to income.^21^ Our finding that ACTC monthly payments were associated with decreased smoking may be explained by payments reducing stress and adverse mental health outcomes, for which there is evidence of in the ACTC literature^22,23,24,25^ and which have a well-known association with tobacco use behaviors.^10^ They were also consistent with a recent randomized clinical trial that showed cash payments reduced mental health and substance-related hospitalizations.^17^

Existing research has shown that ACTC monthly payments were associated with increased spending on items that are essential to childhood well-being and development, including housing,^26^ utilities,^27^ food,^26,27,28^ child-related goods and services,^26,28^ and health care.^28^ Further, research on family outcomes has shown that monthly payments were associated with reduced food insufficiency and material hardship.^29,30,31^ Overall, our findings suggest that these benefits were not outweighed by ACTC monthly payments increasing substance use, and if anything would have been enhanced by the association between ACTC monthly payments and reduced tobacco use.

### Limitations

This study had limitations. First, measurement errors in the survey data could bias results. These include respondents underreporting substance use behaviors to NSDUH,^32,33^ NSDUH not capturing populations for whom substance use is often highest by design (eg, institutionalized and unstably housed individuals), and imprecise measures of tobacco/alcohol use (ranges of cigarettes smoked/alcoholic beverages consumed, which we converted to numbers using midpoints). Second, there were substantive changes to NSDUH in 2020 and 2021 that raise caution about interpreting models that spanned the period from 2018 to 2021, weakening confidence that estimates could be interpreted as causal. These included parts of the survey being administered online in 2020, NSDUH not reporting calendar quarter variables in 2020, and weighting methods changing in 2021. To the best of our ability, we addressed this by restricting our primary analysis to 2021 and providing many ways of assessing preintervention trends (eg, presentation of raw trends in study outcomes and event studies using data from 2018-2021). Finally, quarterly data were also not available in 2022, prohibiting us from evaluating the association between the second half of ACTC payments (which were sent out during the tax season in 2022) and substance use outcomes or observed outcomes after ACTC payments were discontinued.

## Conclusions

The findings of this DiD study suggest that increased income from ACTC monthly payments was not associated with increases in substance use among parents and may have even been associated with reduced parental tobacco use. This evidence does not support policymaker concerns about increased parental substance use outweighing the substantial benefits of ACTC monthly payments to low-income children and families that have been documented in the literature.

## Notes

Supplement 1. eAppendix. Estimating equations eTable 1. Pre- and post-distribution of income for parents vs. non-parents eTable 2. Full Regression Results – Any Substance Use eTable 3. Full Regression Results – Frequency of Substance Use eTable 4. Full Regression Results – Quantity of Substance Use eTable 5. Full Regression Results – Any Substance Use (<200% FPL) eTable 6. Full Regression Results – Frequency of Substance Use (<200% FPL) eTable 7. Full Regression Results – Quantity of Substance Use (<200% FPL) eTable 8. Full Regression Results – Any Substance Use (Continuous Treatment) eTable 9. Full Regression Results – Frequency of Use (Continuous Treatment) eTable 10. Full Regression Results – Quantity of Use (Continuous Treatment) eFigure 1. Trends in the quantity of cigarette and alcohol use in the past 30 days for parents vs. non-parents, 2018-2021 eFigure 2. Changes in distribution of past 30-day cigarette use, parents vs. non-parents, before and after implementation of ACTC monthly payments eFigure 3. Event study plots for any substance use eFigure 4. Event study plots for frequency of substance use eFigure 5. Event study plots for quantity of substance use

Supplement 2. Data sharing statement
