# Error in Supplement 2

**DOI:** 10.1001/jamahealthforum.2025.1249

**Published:** 2025-04-18

In the Original Investigation titled “Advanced Child Tax Credit Monthly Payments and Substance Use Among US Parents,”^1^ published online on January 3, 2025, there was an error in eTable 2 of Supplement 2. The value of tobacco use for “Parent x Post” should be “−0.043,” not “0.043.” This article was corrected online.

## References

[1] Donahoe JT, Brown-Podgorski BL, Gaire S, Krans EE, Jarlenski M. Advanced child tax credit monthly payments and substance use among US parents. JAMA Health Forum. 2025;6(1):e244699. doi:10.1001/jamahealthforum.2024.4699 39752172 PMC11699525

