# Supplementary material for: Advanced Child Tax Credit Monthly Payments and Substance Use Among US Parents
Source: JAMA Health Forum. 2025 Jan 3;6(1):e244699. doi: 10.1001/jamahealthforum.2024.4699 (PMC11699525; doi:10.1001/jamahealthforum.2024.4699)
Supplement: Supplement 1. — eAppendix. Estimating equations eTable 1. Pre- and post-distribution of income for parents vs. non-parents eTable 2. Full Regression Results – Any Substance Use eTable 3. Full Regression Results – Frequency of Substance Use eTable 4. Full Regression Results – Quantity of Substance Use eTable 5. Full Regression Results – Any Substance Use (<200% FPL) eTable 6. Full Regression Results – Frequency of Substance Use (<200% FPL) eTable 7. Full Regression Results – Quantity of Substance Use (<200% FPL) eTable 8. Full Regression Results – Any Substance Use (Continuous Treatment) eTable 9. Full Regression Results – Frequency of Use (Continuous Treatment) eTable 10. Full Regression Results – Quantity of Use (Continuous Treatment) eFigure 1. Trends in the quantity of cigarette and alcohol use in the past 30 days for parents vs. non-parents, 2018-2021 eFigure 2. Changes in distribution of past 30-day cigarette use, parents vs. non-parents, before and after implementation of ACTC monthly payments eFigure 3. Event study plots for any substance use eFigure 4. Event study plots for frequency of substance use eFigure 5. Event study plots for quantity of substance use [file jamahealthforum-e244699-s001.pdf]

Supplemental Online Content

Donahoe JT, Brown-Podgorski BL, Gaire S, Krans EE, Jarlenski M. Advanced child tax credit monthly payments and substance use among US parents. *JAMA Health Forum*. 2024;6(1):e244699. doi:10.1001/jamahealthforum.2024.4699

eAppendix. Estimating equations

eTable 1. Pre- and post-distribution of income for parents vs. non-parents

eTable 2. Full Regression Results – Any Substance Use

eTable 3. Full Regression Results – Frequency of Substance Use

eTable 4. Full Regression Results – Quantity of Substance Use

eTable 5. Full Regression Results – Any Substance Use (<200% FPL)

eTable 6. Full Regression Results – Frequency of Substance Use (<200% FPL)

eTable 7. Full Regression Results – Quantity of Substance Use (<200% FPL)

eTable 8. Full Regression Results – Any Substance Use (Continuous Treatment)

eTable 9. Full Regression Results – Frequency of Use (Continuous Treatment)

eTable 10. Full Regression Results – Quantity of Use (Continuous Treatment)

eFigure 1. Trends in the quantity of cigarette and alcohol use in the past 30 days for parents vs. non-parents, 2018-2021

eFigure 2. Changes in distribution of past 30-day cigarette use, parents vs. non-parents, before and after implementation of ACTC monthly payments

eFigure 3. Event study plots for any substance use

eFigure 4. Event study plots for frequency of substance use

eFigure 5. Event study plots for quantity of substance use

This supplemental material has been provided by the authors to give readers additional information about their work.

© 2024 Donahoe JT. *JAMA Health Forum*.

eAppendix. Estimating Equations

Our primary analysis estimated difference-in-differences regressions of the following form,

$$Y_{i,t} = \beta_1 Parent_i + \beta_2 Post_t + \beta_{12} Parent_i \times Post_t + \gamma X_i + \epsilon_{i,t}$$

with

- $Y_{i,t}$ denoting study outcomes. These included any self-reported use of tobacco, alcohol, cannabis, or illicit substances; the number of days using tobacco, alcohol, cannabis, or illicit substances among people that used these substances; and the number of cigarettes or alcoholic beverages consumed in the past 30 days among people that used these substances.

- $Parent_i$ is a binary variable = 1 if the adult has a dependent child in the home and 0 otherwise.

- $Post_t$ is a binary variable = 1 from July–December 2021, when advance child tax credit monthly payments were in effect.

- X_i is a vector of individual-level covariates. These included age, sex, marital status, education, and race and ethnicity.

In this equation, the estimated association of any advance child tax credit payments with substance use is given by $\hat{\beta}_{12}$. In our sensitivity analysis, we also estimated a variant of this equation that uses the number of dependent children that a person has (as opposed to whether they have any dependent children) as a measure of exposure to advance child tax credit monthly payments (since parents received additional payments per each child).

To examine pre-trends, we also estimated "event-study" versions of equation (1) as follows:

$$Y_{i,t} = \rho Parent_i + \sum_{k=-11;\neq -1}^{1} \pi_r Parent_i \times 1(t + k = 2021Q3) + \delta X_i + \tau_t + v_{i,t}$$

In this equation, $\sum_{k=-11;\neq -1}^{1} \pi_r Parent_i \times 1(t + k = 2021Q3)$ interacts parent status with indicator variables for each period relative to when the advance child tax credit monthly payments went into effect (July – December 2021; 2021Q3). We omit the period just before to normalize differences in outcomes relative to the time-period just before payments were distributed. We used data from 2018-2020. All data from 2020 (for which quarterly data were not available) were treated as one period. Thus, we have 11 pre-ACTC observations (4 in 2018, 4 in 2019, 1 in 2020, and 2 in 2021). The equation also adds in time-period fixed effects.

© 2024 Donahoe JT. *JAMA Health Forum*.

I. Additional Tables and Figures

eTable 1. Pre- and post-distribution of income for parents vs. non-parents

	**Pre-ACTC monthly payments (January – June 2021)**		**Post-ACTC monthly payments (July – December 2021)**	
	**Parents (N = 8,460)**	**Non-parents (n= 12,133)**	**Parents (n = 8,848)**	**Non-parents (N =12,412)**
Mid Income	55422	51272	53887	50685
Income (%)				
Less than $10,000	4.8	8.9	6.0	8.7
$10,000- $19,999	8.4	9.8	8.6	11.0
$20,000- $29,999	7.6	8.1	8.3	8.6
$30,000- $39,999	8.6	8.8	9.0	8.5
$40,000- $49,999	9.2	9.3	9.8	9.4
$50,000- $74,999	16.1	17.0	14.4	14.4
$75,000 or more	45.3	38.1	44.1	39.3

Notes. Table shows the distribution of parents and non-parents across different bins of self-reported income in the NSDUH, before and after implementation of ACTC monthly payments. (%) estimates use survey weights.

© 2024 Donahoe JT. *JAMA Health Forum*.

eTable 2. Full Regression Results – Any Substance Use.

	Probability of any prior 30-day use of:			
	**Tobacco**	**Alcohol**	**Cannabis**	**Illicit Substances**
**Policy variables**				
Parent	0.006	-0.054**	-0.042***	-0.016***
	(0.010)	(0.017)	(0.011)	(0.004)
Post	0.002	-0.009	0.015	-0.002
	(0.009)	(0.012)	(0.012)	(0.004)
Parent × Post	-0.043***	0.010	-0.002	0.008
	(0.011)	(0.022)	(0.015)	(0.006)
**Covariates**				
Age (rel. 18–25)				
26–49	0.154***	0.024*	-0.004	0.025***
	(0.010)	(0.011)	(0.009)	(0.005)
50–64	0.124***	-0.055***	-0.082***	0.018*
	(0.013)	(0.014)	(0.012)	(0.007)
Male gender	0.026***	0.050***	0.037***	0.001
	(0.007)	(0.008)	(0.007)	(0.004)
Race/ethnicity (rel. Hispanic)				
Non-Hispanic Asian	0.039*	-0.208***	-0.023	-0.003
	(0.017)	(0.021)	(0.013)	(0.006)
Non-Hispanic Black	0.044***	-0.009	0.051***	-0.013**
	(0.012)	(0.018)	(0.013)	(0.004)
Non-Hispanic Native American, Alaskan, Hawaiian, or Pacific Islander	0.106***	-0.051	0.134***	0.002
	(0.030)	(0.043)	(0.031)	(0.009)
Non-Hispanic White	0.110***	0.066***	0.071***	0.010**
	(0.011)	(0.013)	(0.008)	(0.003)
Non-Hispanic Multiracial	0.129***	0.048	0.132***	0.017
	(0.026)	(0.031)	(0.024)	(0.010)
Completed college education	-0.183***	0.188***	-0.038***	-0.010***
	(0.006)	(0.010)	(0.007)	(0.002)
Marital Status (rel. married)				
Never married	0.084***	-0.035**	0.100***	0.036***
	(0.012)	(0.011)	(0.009)	(0.006)
Widowed, divorced, or separated	0.175***	-0.005	0.051***	0.028***
	(0.013)	(0.017)	(0.009)	(0.005)
N	41,853	41,853	41,853	41,853

Notes. Data are from the National Survey on Drug Use and Health (NSDUH), among all adults aged 18 to 64. All results use survey weights and are adjusted for clustered sampling. Standard errors are in parentheses. *(**)*** denotes statistical significance at level p<0.05(0.01)0.001.

© 2024 Donahoe JT. *JAMA Health Forum*.

eTable 3. Full Regression Results – Frequency of Substance Use

	Number of days used:			
	**Tobacco**	**Alcohol**	**Cannabis**	**Illicit Substances**
**Policy variables**				
Parent	0.634	-0.356	1.646*	2.128
	(0.723)	(0.376)	(0.640)	(1.784)
Post	0.393	0.235	-0.589	1.336
	(0.535)	(0.302)	(0.688)	(1.283)
Parent × Post	-0.458	-0.735	-0.539	-4.793
	(0.981)	(0.412)	(0.993)	(2.560)
**Covariates**				
Age (rel. 18–25)				
26–49	8.617***	2.014***	2.430***	4.780***
	(0.578)	(0.206)	(0.633)	(0.888)
50–64	10.147***	2.333***	1.062	8.284***
	(0.677)	(0.381)	(0.906)	(1.646)
Male gender	0.634	2.086***	2.042***	-0.699
	(0.405)	(0.243)	(0.412)	(1.132)
Race/ethnicity (rel. Hispanic)				
Non-Hispanic Asian	2.143	0.330	-2.887	0.327
	(1.757)	(0.487)	(1.471)	(2.680)
Non-Hispanic Black	2.109	0.751	1.133	-4.043
	(1.070)	(0.398)	(0.918)	(2.215)
Non-Hispanic Native American, Alaskan, Hawaiian, or Pacific Islander	7.789***	1.108	2.221	-0.146
	(1.164)	(0.933)	(1.898)	(2.874)
Non-Hispanic White	6.036***	2.209***	2.231**	-0.483
	(0.958)	(0.305)	(0.806)	(1.341)
Non-Hispanic Multiracial	5.652***	1.729**	4.215**	2.042
	(1.254)	(0.593)	(1.413)	(2.670)
Completed college education	-3.640***	0.660**	-4.844***	-5.010***
	(0.647)	(0.205)	(0.577)	(1.301)
Marital Status (rel. married)				
Never married	0.339	0.161	1.901**	1.636
	(0.613)	(0.271)	(0.586)	(1.406)
Widowed, divorced, or separated	0.924	0.687	1.429	2.203
	(0.673)	(0.398)	(1.054)	(1.886)
N	6,813	23,319	7,579	1,380

Notes. Data are from the National Survey on Drug Use and Health (NSDUH), among all adults aged 18 to 64 that reported use of each substance. Results use survey weights and are adjusted for clustered sampling. Standard errors are reported in parentheses. *(**)*** denotes statistical significance at level p<0.05(0.01)0.001.

© 2024 Donahoe JT. *JAMA Health Forum*.

eTable 4. Full Regression Results – Quantity of Substance Use

	Quantity of use:	
	**Cigarettes**	**Alcoholic Beverages**
**Policy variables**		
Parent	38.512*	-1.285
	(14.998)	(2.197)
Post	21.358	3.495
	(15.951)	(3.387)
Parent × Post	-46.815*	-6.392
	(23.056)	(4.283)
**Covariates**		
Age (rel. 18–25)		
26–49	160.234***	7.280***
	(12.769)	(1.634)
50–64	223.011***	7.870*
	(21.520)	(3.236)
Male sex	56.301***	14.510***
	(10.644)	(2.277)
Race/ethnicity (rel. Hispanic)		
Non-Hispanic Asian	6.317	-0.558
	(31.756)	(8.674)
Non-Hispanic Black	15.714	-4.514
	(22.329)	(4.775)
Non-Hispanic Native American, Alaskan, Hawaiian, or Pacific Islander	161.513***	16.954
	(38.242)	(12.960)
Non-Hispanic White	189.449***	1.821
	(18.794)	(5.520)
Non-Hispanic Multiracial	127.735***	9.146
	(26.273)	(11.391)
Completed college education	-72.628**	-5.753
	(23.633)	(2.934)
Marital Status (rel. married)		
Never married	-17.164	3.325
	(15.610)	(2.105)
Widowed, divorced, or separated	33.568	12.433**
	(21.118)	(4.590)
N	6,813	23,319

Notes. Data are from the National Survey on Drug Use and Health (NSDUH), among all adults aged 18 to 64 that reported use of each substance. Results use survey weights and are adjusted for clustered sampling. Standard errors are reported in parentheses. *(**)*** denotes statistical significance at level p<0.05(0.01)0.001.

© 2024 Donahoe JT. *JAMA Health Forum*.

eTable 5. Full Regression Results – Any Substance Use (<200% FPL).

	Probability of any prior 30-day use of:			
	**Tobacco**	**Alcohol**	**Cannabis**	**Illicit Substances**
**Policy variables**				
Parent	0.014	-0.025	-0.033	-0.023**
	(0.019)	(0.026)	(0.018)	(0.007)
Post	0.037	-0.020	0.020	0.007
	(0.019)	(0.021)	(0.017)	(0.009)
Parent × Post	-0.061	0.003	0.005	-0.001
	(0.024)	(0.031)	(0.023)	(0.010)
**Covariates**				
Age (rel. 18–25)				
26–49	0.197***	-0.024	-0.013	0.031***
	(0.016)	(0.017)	(0.013)	(0.007)
50–64	0.173***	-0.138***	-0.100***	0.019
	(0.028)	(0.022)	(0.019)	(0.011)
Male gender	0.057***	0.051***	0.068***	0.007
	(0.012)	(0.012)	(0.012)	(0.007)
Race/ethnicity (rel. Hispanic)				
Non-Hispanic Asian	0.010	-0.191***	-0.005	0.001
	(0.021)	(0.034)	(0.021)	(0.011)
Non-Hispanic Black	0.066**	0.001	0.078***	-0.012
	(0.019)	(0.026)	(0.015)	(0.007)
Non-Hispanic Native American, Alaskan, Hawaiian, or Pacific Islander	0.152**	-0.053	0.188***	0.003
	(0.044)	(0.049)	(0.040)	(0.013)
Non-Hispanic White	0.218***	0.035	0.132***	0.024**
	(0.018)	(0.023)	(0.013)	(0.007)
Non-Hispanic Multiracial	0.206***	0.054	0.184***	0.045*
	(0.042)	(0.046)	(0.038)	(0.020)
Completed college education	-0.174***	0.132***	-0.039*	-0.018**
	(0.013)	(0.021)	(0.016)	(0.006)
Marital Status (rel. married)				
Never married	0.073**	0.051*	0.082***	0.030***
	(0.023)	(0.021)	(0.015)	(0.007)
Widowed, divorced, or separated	0.165***	0.080**	0.043**	0.028**
	(0.021)	(0.024)	(0.014)	(0.008)
N	15,324	15,324	15,324	15,324

Notes. Data are from the National Survey on Drug Use and Health (NSDUH), among all adults aged 18 to 64. All results use survey weights and are adjusted for clustered sampling. Standard errors are in parentheses. *(**)*** denotes statistical significance at level p<0.05(0.01)0.001.

© 2024 Donahoe JT. *JAMA Health Forum*.

eTable 6. Full Regression Results – Frequency of Substance Use (<200% FPL)

	Number of days used:			
	**Tobacco**	**Alcohol**	**Cannabis**	**Illicit Substances**
**Policy variables**				
Parent	0.619	-1.098	1.151	-0.534
	(0.971)	(0.564)	(1.035)	(2.746)
Post	0.652	0.070	-0.856	0.881
	(0.785)	(0.573)	(1.149)	(2.374)
Parent × Post	-0.073	-0.158	1.105	-1.430
	(1.103)	(0.702)	(1.595)	(4.023)
**Covariates**				
Age (rel. 18–25)				
26–49	8.299***	1.947***	2.945***	5.859***
	(0.780)	(0.426)	(0.755)	(1.411)
50–64	8.796***	1.325	1.849	8.337**
	(1.203)	(0.828)	(1.263)	(2.620)
Male gender	0.846	1.470***	2.478***	0.883
	(0.572)	(0.372)	(0.695)	(1.635)
Race/ethnicity (rel. Hispanic)				
Non-Hispanic Asian	1.081	-0.639	-5.088*	5.331
	(2.675)	(0.911)	(2.065)	(4.198)
Non-Hispanic Black	2.630	1.618*	0.898	-5.240
	(1.452)	(0.656)	(1.363)	(3.046)
Non-Hispanic Native American, Alaskan, Hawaiian, or Pacific Islander	8.163***	1.501	-0.048	-1.277
	(1.430)	(1.402)	(2.348)	(5.031)
Non-Hispanic White	7.289***	2.363***	3.268**	-0.779
	(1.309)	(0.570)	(1.170)	(3.022)
Non-Hispanic Multiracial	6.370***	0.854	4.297*	-1.975
	(1.810)	(0.772)	(1.974)	(4.161)
Completed college education	-2.137	0.045	-2.205*	-1.531
	(1.148)	(0.501)	(1.019)	(1.993)
Marital Status (rel. married)				
Never married	0.283	0.677	0.427	-1.952
	(0.982)	(0.512)	(0.907)	(2.910)
Widowed, divorced, or separated	0.885	2.159**	-0.197	-0.849
	(1.031)	(0.760)	(1.416)	(3.084)
N	3,705	6,980	3,364	678

Notes. Data are from the National Survey on Drug Use and Health (NSDUH), among all adults aged 18 to 64 that reported use of each substance. Results use survey weights and are adjusted for clustered sampling. Standard errors are reported in parentheses. *(**)*** denotes statistical significance at level p<0.05(0.01)0.001.

© 2024 Donahoe JT. *JAMA Health Forum*.

eTable 7. Full Regression Results – Quantity of Substance Use (<200% FPL)

	Quantity of use:	
	**Cigarettes**	**Alcoholic Beverages**
**Policy variables**		
Parent	37.213	-5.896
	(18.719)	(4.934)
Post	30.491	0.760
	(26.262)	(5.572)
Parent × Post	-47.521	-2.661
	(35.774)	(7.725)
**Covariates**		
Age (rel. 18–25)		
26–49	147.620***	12.293***
	(17.005)	(2.721)
50–64	200.982***	8.765
	(35.119)	(6.645)
Male sex	63.872***	13.763***
	(13.502)	(3.374)
Race/ethnicity (rel. Hispanic)		
Non-Hispanic Asian	40.118	7.538
	(46.197)	(13.692)
Non-Hispanic Black	44.286	1.541
	(29.282)	(3.946)
Non-Hispanic Native American, Alaskan, Hawaiian, or Pacific Islander	174.072***	31.404
	(48.969)	(22.259)
Non-Hispanic White	214.248***	10.626*
	(28.531)	(4.809)
Non-Hispanic Multiracial	128.689***	34.200
	(33.191)	(27.415)
Completed college education	-83.206**	-9.401**
	(28.400)	(3.050)
Marital Status (rel. married)		
Never married	-22.248	4.973
	(19.787)	(3.804)
Widowed, divorced, or separated	37.248	20.150**
	(24.333)	(6.995)
N	3,705	6,980

Notes. Data are from the National Survey on Drug Use and Health (NSDUH), among all adults aged 18 to 64 that reported use of each substance. Results use survey weights and are adjusted for clustered sampling. Standard errors are reported in parentheses. *(**)*** denotes statistical significance at level p<0.05(0.01)0.001.

© 2024 Donahoe JT. *JAMA Health Forum*.

eTable 8. Full Regression Results – Any Substance Use (Continuous Treatment).

	Probability of any prior 30-day use of:			
	**Tobacco**	**Alcohol**	**Cannabis**	**Illicit Substances**
**Policy variables**				
# Children	0.004	-0.031***	-0.022***	-0.007***
	(0.005)	(0.007)	(0.005)	(0.002)
Post	0.013	-0.008	0.012	-0.002
	(0.009)	(0.011)	(0.011)	(0.004)
# Children × Post	-0.015*	0.004	0.003	0.004
	(0.007)	(0.009)	(0.008)	(0.002)
**Covariates**				
Age (rel. 18–25)				
26–49	0.154***	0.027*	-0.002	0.026***
	(0.010)	(0.011)	(0.009)	(0.005)
50–64	0.126***	-0.056***	-0.079***	0.019*
	(0.013)	(0.014)	(0.012)	(0.007)
Male gender	0.027***	0.050***	0.037***	0.001
	(0.007)	(0.008)	(0.007)	(0.004)
Race/ethnicity (rel. Hispanic)				
Non-Hispanic Asian	0.039*	-0.209***	-0.024	-0.003
	(0.017)	(0.021)	(0.013)	(0.006)
Non-Hispanic Black	0.044***	-0.010	0.051***	-0.013**
	(0.012)	(0.018)	(0.013)	(0.004)
Non-Hispanic Native American, Alaskan, Hawaiian, or Pacific Islander	0.106***	-0.050	0.136***	0.003
	(0.030)	(0.043)	(0.031)	(0.009)
Non-Hispanic White	0.111***	0.065***	0.071***	0.010**
	(0.011)	(0.013)	(0.008)	(0.003)
Non-Hispanic Multiracial	0.129***	0.049	0.133***	0.018
	(0.026)	(0.031)	(0.024)	(0.010)
Completed college education	-0.183***	0.187***	-0.038***	-0.010***
	(0.006)	(0.010)	(0.008)	(0.002)
Marital Status (rel. married)				
Never married	0.087***	-0.038**	0.101***	0.036***
	(0.012)	(0.012)	(0.009)	(0.006)
Widowed, divorced, or separated	0.176***	-0.006	0.051***	0.029***
	(0.013)	(0.018)	(0.009)	(0.005)
N	41,853	41,853	41,853	41,853

Notes. Data are from the National Survey on Drug Use and Health (NSDUH), among all adults aged 18 to 64. All results use survey weights and are adjusted for clustered sampling. Standard errors are in parentheses. *(**)*** denotes statistical significance at level p<0.05(0.01)0.001.

© 2024 Donahoe JT. *JAMA Health Forum*.

eTable 9. Full Regression Results – Frequency of Use (Continuous Treatment)

	Number of days used:			
	**Tobacco**	**Alcohol**	**Cannabis**	**Illicit Substances**
**Policy variables**				
# Children	0.284	-0.057	0.770*	1.443
	(0.351)	(0.209)	(0.344)	(0.935)
Post	0.255	0.243	-0.625	0.945
	(0.509)	(0.295)	(0.669)	(1.209)
# Children × Post	-0.041	-0.433*	-0.272	-2.077
	(0.467)	(0.215)	(0.477)	(1.280)
**Covariates**				
Age (rel. 18–25)				
26–49	8.592***	2.017***	2.375***	4.663***
	(0.579)	(0.206)	(0.636)	(0.892)
50–64	10.172***	2.408***	0.950	8.429***
	(0.691)	(0.386)	(0.882)	(1.674)
Male gender	0.646	2.104***	2.017***	-0.592
	(0.399)	(0.242)	(0.412)	(1.131)
Race/ethnicity (rel. Hispanic)				
Non-Hispanic Asian	2.180	0.326	-2.890	0.512
	(1.750)	(0.484)	(1.455)	(2.677)
Non-Hispanic Black	2.118	0.744	1.120	-4.078
	(1.069)	(0.397)	(0.929)	(2.243)
Non-Hispanic Native American, Alaskan, Hawaiian, or Pacific Islander	7.754***	1.114	2.158	0.013
	(1.175)	(0.938)	(1.907)	(2.836)
Non-Hispanic White	6.050***	2.224***	2.216**	-0.467
	(0.958)	(0.307)	(0.802)	(1.375)
Non-Hispanic Multiracial	5.644***	1.759**	4.182**	2.131
	(1.250)	(0.597)	(1.424)	(2.687)
Completed college education	-3.627***	0.671**	-4.888***	-4.976***
	(0.648)	(0.203)	(0.574)	(1.332)
Marital Status (rel. married)				
Never married	0.371	0.238	1.859**	1.923
	(0.609)	(0.276)	(0.581)	(1.396)
Widowed, divorced, or separated	0.946	0.711	1.443	2.394
	(0.676)	(0.399)	(1.050)	(1.922)
N	6,813	23,319	7,579	1,380

Notes. Data are from the National Survey on Drug Use and Health (NSDUH), among all adults aged 18 to 64 that reported use of each substance. Results use survey weights and are adjusted for clustered sampling. Standard errors are reported in parentheses. *(**)*** denotes statistical significance at level p<0.05(0.01)0.001.

© 2024 Donahoe JT. *JAMA Health Forum*.

eTable 10. Full Regression Results – Quantity of Use (Continuous Treatment)

	Quantity of use:	
	**Cigarettes**	**Alcoholic Beverages**
**Policy variables**		
# Children	15.590*	-0.031
	(6.731)	(1.303)
Post	14.597	3.063
	(15.454)	(3.058)
# Children × Post	-15.493	-3.018
	(10.216)	(2.070)
**Covariates**		
Age (rel. 18–25)		
26–49	159.581***	7.246***
	(12.876)	(1.616)
50–64	222.790***	8.411*
	(21.343)	(3.338)
Male sex	56.592***	14.654***
	(10.580)	(2.305)
Race/ethnicity (rel. Hispanic)		
Non-Hispanic Asian	7.188	-0.549
	(31.948)	(8.720)
Non-Hispanic Black	15.607	-4.552
	(22.532)	(4.786)
Non-Hispanic Native American, Alaskan, Hawaiian, or Pacific Islander	159.837***	17.038
	(38.874)	(12.973)
Non-Hispanic White	189.615***	1.935
	(19.092)	(5.523)
Non-Hispanic Multiracial	126.987***	9.331
	(26.480)	(11.396)
Completed college education	-72.815**	-5.666
	(23.425)	(2.927)
Marital Status (rel. married)		
Never married	-17.169	3.909
	(15.633)	(2.151)
Widowed, divorced, or separated	33.327	12.614**
	(21.458)	(4.624)
N	6,813	23,319

Notes. Data are from the National Survey on Drug Use and Health (NSDUH), among all adults aged 18 to 64 that reported use of each substance. Results use survey weights and are adjusted for clustered sampling. Standard errors are reported in parentheses. *(**)*** denotes statistical significance at level p<0.05(0.01)0.001.

© 2024 Donahoe JT. *JAMA Health Forum*.

II. Additional Figures

eFigure 1. Trends in the quantity of cigarette and alcohol use in the past 30 days for parents vs. non-parents, 2018-2021.

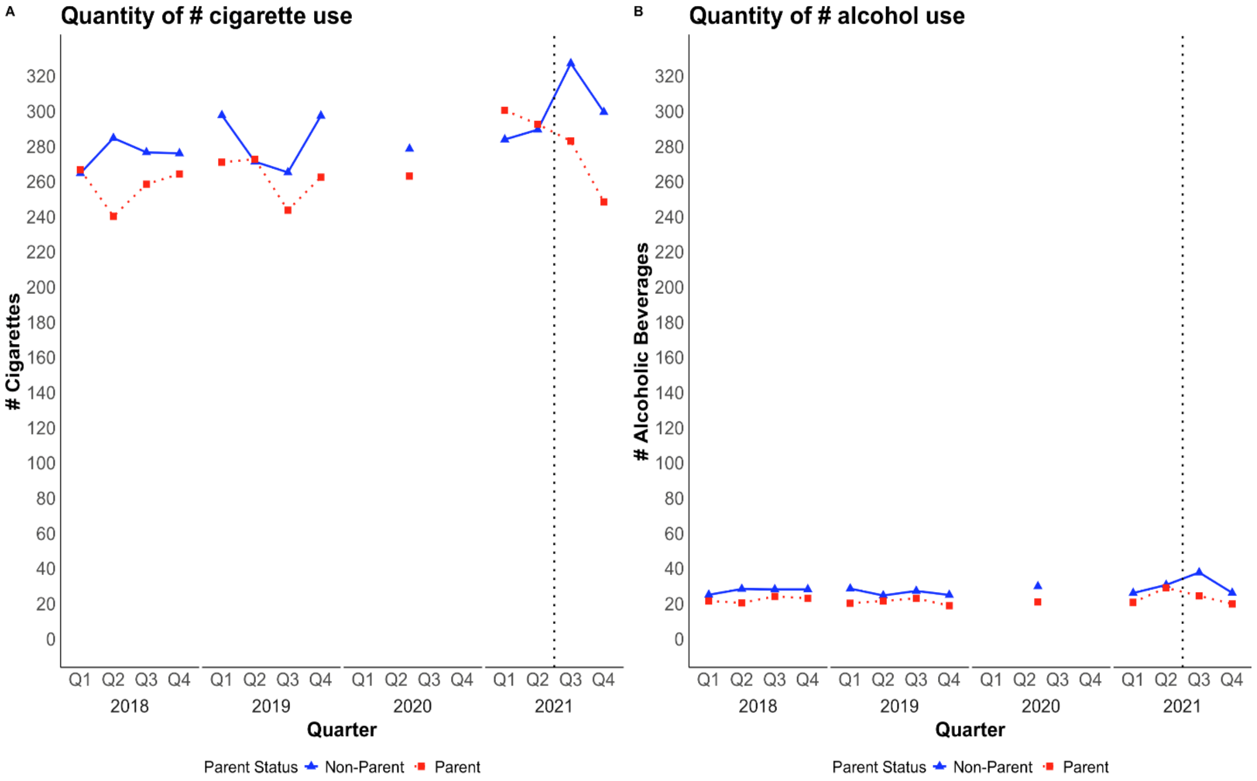

Notes. Data are from the National Survey on Drug Use and Health (NSDUH), among all adults aged 18 to 64 who reported use of cigarettes (A) or alcohol (B), from 2018-2021. The vertical line indicates when advance child tax credit monthly payments were sent out from July–December 2021. Quarterly data were not available for 2020.

© 2024 Donahoe JT. *JAMA Health Forum*.

eFigure 2. Changes in distribution of past 30-day cigarette use, parents vs. non-parents, before and after implementation of ACTC monthly payments.

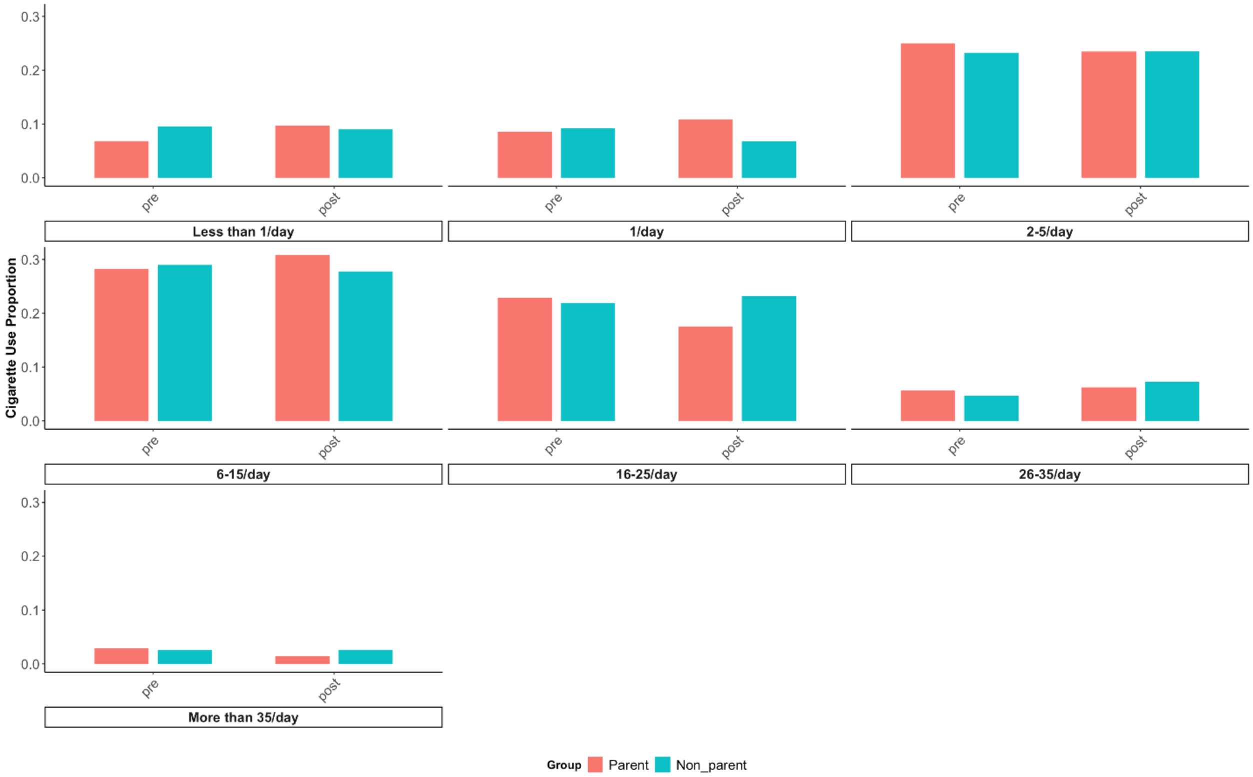

Notes. Results show the proportion of parents and non-parents who report smoking different quantities of cigarettes per day when the smoke in 2021, before and after implementation of the ACTC monthly payments. Results use survey weights.

© 2024 Donahoe JT. *JAMA Health Forum*.

eFigure 3. Event study plots for any substance use

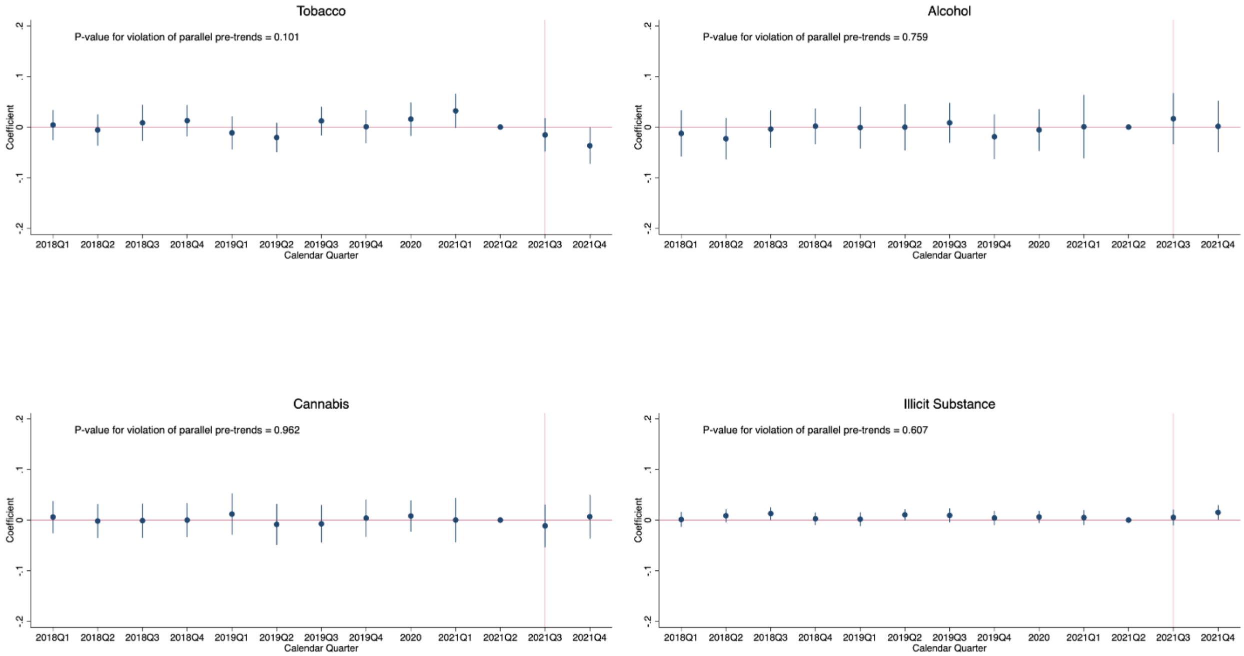

Notes. Results show event study coefficients (interaction between parental status and quarters relative to ACTC monthly payments) for each of the outcomes shown. The vertical line denotes when ACTC monthly payments went into effect (2021 Q3). Results use survey weights and are adjusted for clustered sampling.

© 2024 Donahoe JT. *JAMA Health Forum*.

eFigure 4. Event study plots for frequency of substance use

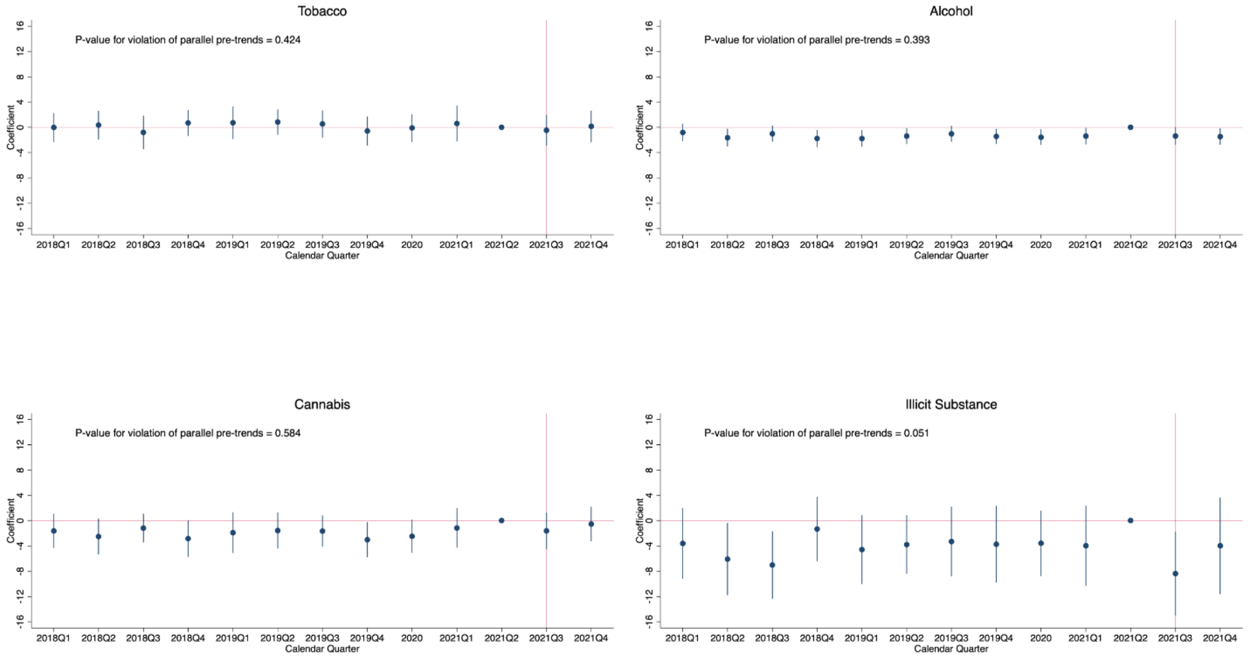

Notes. Results show event study coefficients (interaction between parental status and quarters relative to ACTC monthly payments) for each of the outcomes shown. The vertical line denotes when ACTC monthly payments went into effect (2021 Q3). Results use survey weights and are adjusted for clustered sampling.

© 2024 Donahoe JT. *JAMA Health Forum*.

eFigure 5. Event study plots for quantity of substance use

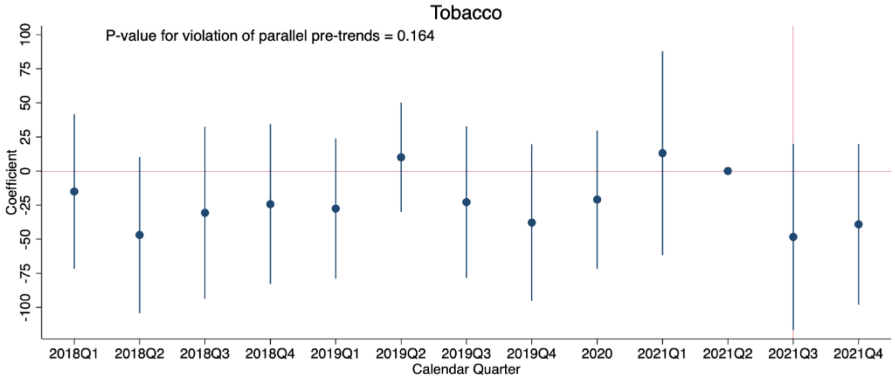

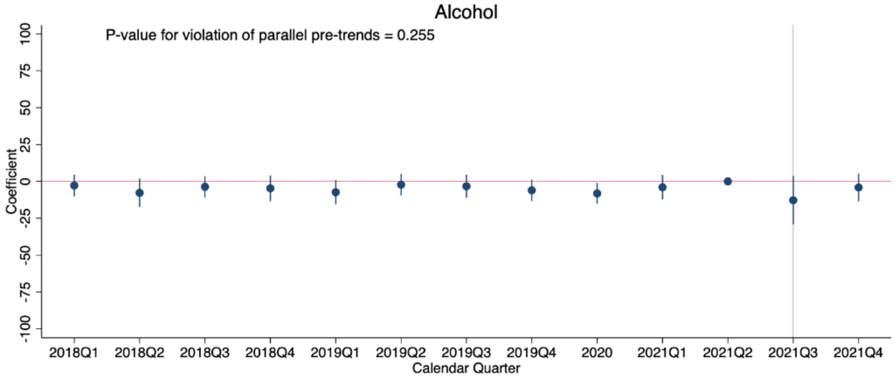

Notes. Results show event study coefficients (interaction between parental status and quarters relative to ACTC monthly payments) for each of the outcomes shown. The vertical line denotes when ACTC monthly payments went into effect (2021 Q3). Results use survey weights and are adjusted for clustered sampling.

© 2024 Donahoe JT. *JAMA Health Forum*.
