# Supplementary material for: Advanced Child Tax Credit Monthly Payments and Substance Use Among US Parents
Source: JAMA Health Forum. 2025 Jan 3;6(1):e244699. doi: 10.1001/jamahealthforum.2024.4699 (PMC11699525; doi:10.1001/jamahealthforum.2024.4699)
Supplement: Supplement 2. — Data sharing statement [file jamahealthforum-e244699-s002.pdf]

Data Sharing Statement

Donahoe. Advanced Child Tax Credit Monthly Payments and Substance Use Among US Parents. *JAMA Health Forum*. Published January 03, 2025.
doi:10.1001/jamahealthforum.2024.4699

Data
Data available: Yes
Data types: Deidentified participant data
How to access data: Data are from the publicly available National Survey on Drug Use and Health. We will make code to replicate our analysis available on the first author's website with publication.
When available: With publication

Supporting Documents
Document types: Statistical/analytic code
How to access documents: We will make code to replicate our analysis available on the first author's website with publication.
When available: With publication

Additional Information
Who can access the data: Anyone requesting the data.
Types of analyses: Any purpose.
Mechanisms of data availability: Any mechanism.
Any additional restrictions: N/A
